# Multi-Mode Model Predictive Control Approach for Steel Billets Reheating Furnaces

**DOI:** 10.3390/s23083966

**Published:** 2023-04-13

**Authors:** Silvia Maria Zanoli, Crescenzo Pepe, Lorenzo Orlietti

**Affiliations:** 1 Dipartimento di Ingegneria dell’Informazione, Università Politecnica delle Marche, Via Brecce Bianche 12, 60131 Ancona, Italy; c.pepe@univpm.it; 2 Alperia Green Future, Via Dodiciville 8, 39100 Bolzano, Italy

**Keywords:** steel industry, reheating furnace, level 2 advanced process control, model predictive control, energy efficiency

## Abstract

In this paper, a unified level 2 Advanced Process Control system for steel billets reheating furnaces is proposed. The system is capable of managing all process conditions that can occur in different types of furnaces, e.g., walking beam and pusher type. A multi-mode Model Predictive Control approach is proposed together with a virtual sensor and a control mode selector. The virtual sensor provides billet tracking, together with updated process and billet information; the control mode selector module defines online the best control mode to be applied. The control mode selector uses a tailored activation matrix and, in each control mode, a different subset of controlled variables and specifications are considered. All furnace conditions (production, planned/unplanned shutdowns/downtimes, and restarts) are managed and optimized. The reliability of the proposed approach is proven by the different installations in various European steel industries. Significant energy efficiency and process control results were obtained after the commissioning of the designed system on the real plants, replacing operators’ manual conduction and/or previous level 2 systems control.

## 1. Introduction

The steel industry provides essential materials to modern society. The annual consumption of steel is growing due to global development. Due to the central role of fuel in ironmaking and steelmaking, carbon dioxide (CO_2_) emissions are large, corresponding to approximately 7% of the total anthropogenic CO_2_ emissions. In this context, the steel industry must reduce its CO_2_ emissions as much as possible by improving and developing the process towards carbon neutrality [1,2,3].

In a steel plant, different heating processes take place, e.g., the ones related to blast furnaces, electric arc furnaces, and reheating furnaces. Reheating furnaces are used in the steel industry for reheating and heat treatment of semi-finished products (e.g., billets, slabs) before they can undergo forming/plastic deformation processes, e.g., hot rolling in a mill. The reheating process is a critical process that consumes large amounts of energy in terms of fuel, incurring considerable costs and directly affecting product quality [4,5].

Hardware and software interventions can be proposed in the steel industry reheating furnaces/rolling mills for CO_2_ emissions reduction. Data selection, acquisition, storage, and analysis play a fundamental role in this context: the Industry 4.0 framework can provide useful and efficient methods for these purposes [6,7,8]. Through hardware interventions, some aspects of the production chain are modified; an example is constituted by the installation of a huge number of “micro-mills” with induction furnaces [9,10]. An additional hardware modification solution is represented by process improvement through heat-mass transfer and thermal efficiency calculation [11]. On the other hand, tailored software solutions can be designed in order to optimize the already existing plants, without requiring significant hardware modifications. In this context, traditional control systems, Advanced Process Control (APC) systems, and high-level optimization systems can be utilized [3,12]. These systems in the steel industry reheating furnaces can be located at levels 1, 2, and 3 of the automation hierarchy [13]. Different researchers, engineers, and practitioners proposed various types of solutions located at the mentioned levels of the automation hierarchy.

Level 1 control solutions compute the power to be injected in each furnace zone. The power consists of gas demands that must be provided to the considered furnace zone together with the needed air flow rate in order to respect the defined stoichiometric ratio. The computation takes into account the required zone temperature setpoint (provided by level 2) in order to track it. Examples of level 1 control solutions were reported in [13,14,15,16,17,18,19]. A distributed Model Predictive Control (MPC) strategy is proposed in [13], using a model which considers the coupling effect between zones. A comparison between the proposed control strategy and a standard Proportional–Integral–Derivative (PID)-based controller is provided through tailored experiments. In [14], the design of a hybrid intelligent controller based on condition identification for the combustion process of a heating furnace is proposed, considering stable and fluctuating working conditions. A fuzzy controller is designed to improve the control accuracy of furnace temperature under stable working conditions, and an expert controller is used to adjust the temperature rapidly under fluctuating working conditions. The proposed method was tested on an industrial site. In [15], an optimal design of the furnace combustion control loop via the cross-limiting control strategy is proposed in order to improve a traditional furnace temperature-combustion cascade control system. Energy consumption and environmental pollution are used as indicators for performance evaluation. In [16], a cascade control system of temperature and flow with double cross-limited control is proposed. A design method of an intelligent temperature controller based on an adaptive neural fuzzy inference system is provided. The designed controller is tested through simulations. A modification of the Smith predictor scheme is proposed in [17], combined with a gain-scheduled fuzzy block strategy, for controlling the soaking zone temperature in a steel slab-reheating furnace. A soaking zone temperature dynamic model is obtained from an identification procedure, resulting in a second order plus time-delay transfer function, where the dominant time delay varies with respect to the slab thickness. The performance of the proposed method is compared against the filtered Smith Predictor and the classic one. In [18], the use of a Generalized Predictive Control (GPC) is proposed and a comparison between a Smith Predictor, Fuzzy Control, and GPC is designed in order to find the best control technique that results in the shortest set-up time while optimizing fuel consumption. The designed control approaches were tested through simulations taking into account different operating conditions. In [19], the time-domain coupling relationship of the burner layout, injection angle, and air–gas ratio was simulated to analyze the influence of the flow field on the transient temperature field and the smoke emission in a regenerative pusher reheating furnace. The formulated model improves the prediction accuracy of the temperature field, which could avoid some problems, such as insufficient burning, over-burning, and overheating.

Level 3 systems refer to production scheduling and to the computation of the temperature profile of each piece to be reheated/rolled. Systems located at level 3 are reported in [20,21,22,23,24,25]. A review of planning and scheduling methods for hot rolling mills in steel production is reported in [20]. Diversity in optimization methods, constraints incorporated in the analysis and level of abstraction, and data availability are considered in the study. In [21], an algorithm for modeling electricity and natural gas consumption in a walking furnace is proposed, using artificial intelligence and simulation methods and considering the length of the rolling campaign and the established rolling program. A proposal for a set of minimum requirements characterizing the Best Available Techniques for beam furnaces intended for hot rolling is achieved. A mathematical model for a reheating furnace is proposed in [22], by considering the efficiency–temperature relationship of the furnace. The model allows us to identify the most proper optimization of the temperature–time relation in different productive situations. It is thus capable of guaranteeing the most energy-efficient reheating operations by preserving logistics performances. In [23], the integrated scheduling of the reheating furnace and hot rolling is investigated in view of multi-objective optimization. A mixed-integer programming model with two objectives is formulated for this problem, and a multiobjective differential evolution algorithm is developed to solve this model. The developed methods are tested through simulated data. In [24], the problem of synchronized reheating furnaces and hot rolling mills is investigated. The task is to set the feed-in and drop-out times of slabs to different types of reheat furnaces to reduce the dispensable energy consumption and increase the production rate of the whole process. In [25], a data mining approach for multi-influence factors on billet gas consumption in a reheating furnace is proposed. The multi-influence factors data mining model includes different steps, e.g., an apportionment model. Some measures which could improve the residence time and loading temperature are achieved.

With regard to level 2, it usually determines the temperature setpoint for each zone of the furnace according to the scheduling of products, their desired discharged temperature, and the instantaneous thermal state of the furnace [26]. Reheating furnaces are characterized by a high number of variables to be considered through a real-time operation. Furthermore, the absence of direct temperature measurements for the semi-finished products within the furnace, together with the nonlinear dynamics, the presence of many constraints on the inputs and outputs, and many process disturbances result in a nontrivial level 2 control and optimization problem [4]. In [4], the advantages of using a model-based control/optimization framework for steel reheating furnaces are discussed; in particular, a mathematical model of a continuous slab reheating furnace is proposed together with the related control system. The developed package is first tested through accurate simulation experiments and then implemented and used on an industrial slab reheating furnace. In [11], an analysis to explore possibilities for the improvement in the energy efficiency of an operating natural gas-fired reheating furnace is proposed. As a result of these investigations, energy-saving opportunities were ascertained. For example, a solution for unforeseen mill delays is proposed, improving the previous delay and shutdown logics related to the zone temperature setpoints. In [27], a genetic algorithm approach is proposed in order to obtain a billet reheating furnace model and to include it in a nonlinear optimization problem. The goal is represented by the minimization of fuel costs while satisfying a desired discharge temperature; simulation results are shown. In [28], a double model slab tracking system for a walking beam continuous reheating furnace is developed. A ternary model is used to describe the heating process in the furnace. The model is validated through experimental data obtained through two trailing thermocouple experiments and then is exploited as a simulator for control purposes. In [29], a first-principles mathematical model for a continuous reheating furnace of steel slabs is proposed; a nonlinear model predictive controller is then designed that defines local furnace temperatures so that the slabs reach their desired final temperatures. The proposed system is tested through a real industrial application. A multi-objective optimization problem for a large-scale bloom reheating furnace is presented in [30]. For a given production configuration, a genetic algorithm is used to determine an optimal temperature trajectory of the bloom so as to minimize an appropriate cost function defined by a set of fuzzy rules. These rules take into account different trade-offs between the bloom’s desired discharge temperature, temperature uniformity, and specific fuel consumption. The proposed system is simulated under different scenarios. In [31], a general structure of an optimal control system for heating billets in a reheating furnace before rolling is given. The presented control system shows the relationship of individual subsystems, each of which performs a strictly defined function. An information exchange procedure between the different sub-processes is given and this allows for the creation of optimal heating modes, taking into account all aspects that arise during the heating process. In [32], a dynamic optimizer for temperature control of steel slabs in a continuous reheating furnace is proposed. A hierarchical control structure is designed based on a continuous time-switched nonlinear model: furnace zone temperatures are used as intermediate control variables. Constraints on system states and control variables are considered by penalty terms in the cost function and saturation functions. The capabilities of the proposed method are demonstrated through an example problem. In [33], a hierarchical control approach is proposed. Heat inputs for each furnace slab are computed based on a discrete-time nonlinear model. Subsequently, reference trajectories of furnace temperatures are planned through the solution of a quadratic program. An example problem shows the feasibility and the limitations of the approach. In [34], a two-dimensional mathematical heat transfer model for the prediction of the temperature history of steel slabs is proposed. The objective is to reach an optimal heating pattern for the slabs with minimum energy consumption in a walking-beam type reheating furnace. A simplified conjugate-gradient method combined with a shooting method represents the algorithm for the solution of the obtained optimization problem aimed at obtaining optimal zone temperature setpoints. In [35], a multi-objective optimization method is proposed for reheating the furnace’s temperature setting based on a particle swarm optimization algorithm. A 2D model of the finite difference scheme, in which the thickness and width were unequally partitioned, is developed. A multi-objective optimization function of the temperature setting, from which energy consumption, oxidation, and burning loss should be minimized, is established. In [36], the slab discharge temperature, the slab temperature uniformity, and the specific fuel consumption are included in a function value-based multi-objective optimization problem. Hooke-Jeeves’ direct search algorithm was used to minimize the objective functions under a series of production rates. The optimized setpoint temperatures were further used to construct an artificial neural network of setpoint temperature in each control zone to update the setpoint temperatures when the reheating furnace encounters a production rate change. In [37], a Computational Fluid Dynamics (CFD) model of an industrial roller-hearth reheating furnace with radiant tubes is developed. The movement of the slab on the rollers is performed using dynamic mesh. The CFD model is compared to measurements and the original heating process in production is optimized by the CFD model.

This paper proposes a unified level 2 APC framework for steel billets reheating furnaces. The paper aims to provide holistic knobs and solutions for the assessment of level 2 APC methods for different types of steel industry billets reheating furnaces, e.g., walking beam and pusher type. To the best of the authors’ knowledge, a unified level 2 control framework for steel billets reheating furnaces capable to manage all process conditions and equipped with the needed customization procedures for each different case study is not present in the literature. The main parts which characterize the proposed APC system are: a billets’ virtual sensor, a control mode selector, and a multi-mode MPC block. The innovative features of the proposed approach are:

- a rolling mill stand absorption model tailored for control purposes;
- an adaptation policy of the coefficients associated with the model of billet features;
- the set of adopted Controlled Variables (CVs);
- a double control option for the billet head–tail temperature difference;
- a strategy for computing billet temperature control specifications at different positions at the furnace exit;
- a multi-mode MPC approach that handles all process conditions and can be used in different furnace types (e.g., walking beam and pusher type) and regardless of the adopted billets’ model;
- a control mode selector which online activates the best MPC mode in each process condition;
- a billet temperature model linearization approach that balances the accuracy of control and the burden of optimization problem solver.

The control and energy-saving results achieved with the different field implementations confirmed the validity of the proposed approach. Projects which include the implementation on the real process and are designed as lasting control applications and not as temporary tests are not widespread. The field application of an APC system designed and tested through virtual environment simulations requires significant reliability and robustness features in order to bridge the gap between simulations and field application.

The paper is organized as follows: Section 2 reports the material and methods, providing: the steel billets reheating furnaces’ description (process main features, process automation, and level 2 control and optimization specifications), the proposed level 2 APC system (data structures, billets’ virtual sensor, APC variables and specifications, furnace condition detection, and multi-mode MPC definition) and the multi-mode MPC formulation (general formulation, control modes characterization). Section 3 reports the results and discussion, focusing on modelization, control, and energy efficiency aspects. The conclusions are summarized in Section 4, together with some ideas for future work.

## 2. Materials and Methods

### 2.1. Steel Billets Reheating Furnaces

In this section, steel billets reheating furnaces’ main features are described, together with the process automation and the control and optimization specifications [38,39]. In addition, the case study that will be considered for the illustration of some results achieved is reported.

#### 2.1.1. Process Description

**Figure 1:**
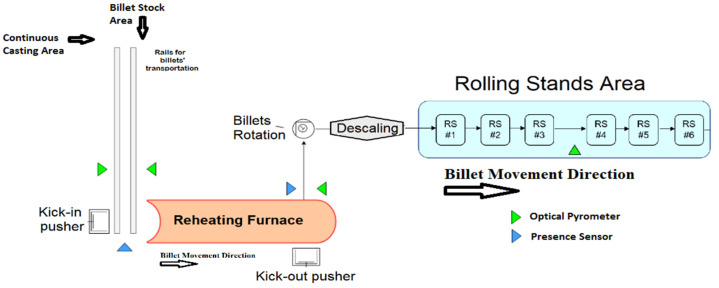
Example of the steel industry subpart related to a billet reheating furnace.

Billets reheating furnaces are part of hot rolling mill units. As shown in Figure 1, they are typically located between the continuous casting (and the related sorting lines) and the rolling mill stands. Billets are semi-finished products that are obtained through the casting of raw materials and their subsequent solidification in tailored molds. Billets can be characterized by a square section, e.g., 0.14 m × 0.14 m or 0.15 m × 0.15 m, and different lengths, e.g., 9 m or 12 m. The steel composition of every single billet can be different. Downstream of the continuous casting, there may be one or more sorting lines to direct billets straight to the furnace’s entrance or to storage areas. In this way, different production or stock lines can supply the reheating furnace (see Figure 1). For this reason, the furnace inlet temperature of every single billet can be different; an example of an inlet temperature range is from 30 °C to 900 °C.

**Figure 2:**
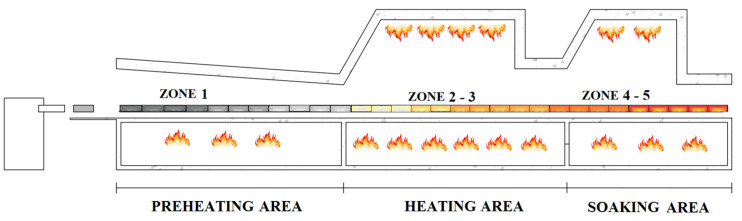
Schematic representation of a steel industry billets reheating furnace.

Billets can be charged in the furnace according to different configurations, e.g., individually or in pairs. The charging action can be performed by kick-in pushers (see Figure 1). Movement procedures can be different and characterize the furnace typology: for example, in a pusher-type reheating furnace, billets are moved along the furnace through the action of kick-in pushers and no empty spaces are present between billets within the furnace. During their movement, billets pass through different furnace areas (see Figure 2), composed of one or more zones: Preheating Area, Heating Area, and Soaking Area. Along their path along the furnace, billets pass through areas of increasing temperatures and are subject to conduction, convection, and radiation phenomena. In the Preheating Area, billets are subjected to a preheating phase that is performed thanks to the hot gasses from the downstream areas and, eventually, by the combustion reactions triggered in the related zones. A critical phase for billets’ reheating takes place in the Heating Area, where major combustion reactions are triggered. The billets heating process ends with temperature equalization in the Soaking Area (see Figure 2). Typically, this area is characterized by asymmetric heating management so that two different zones, perpendicularly to the furnace’s longitudinal axis, characterized by different temperatures, are created. In this way, a different reheating can be ensured for the head and the tail of the billets. Nearby the furnace inlet, a heat exchanger, called a smoke exchanger, preheats air supplied to burners, through a suitable treatment of combustion smokes. The combustion smokes are ejected through a chimney.

The path of the billets within the furnace can be continuous at a fixed/time-varying production rate and/or subjected to planned/unplanned shutdowns/downtimes and restarts. Therefore, the furnace movement time (and the furnace production rate) can vary according to the plant production planning and scheduling and to the subsequent rolling phase requirements; for example, finished products changes or delays in the rolling mill (e.g., due to inter-locking) may result in a temporary stop or slowdown of the billets movement along the furnace. Billets’ discharge from the furnace can be performed through tailored discharge devices, e.g., kick-out pushers (see Figure 1). After their exit, billets transit on a rotating roller track toward a descaling device which reduces the amount of scale on the billet’s surface, e.g., using water at high pressure (see Figure 1) [40,41].

Subsequently, billets enter the rolling mill stands (see Figure 1), where they are moved by transport rollers and are subjected to a forming/plastic deformation process in order to obtain the final product, e.g., rods or tube rounds. The billets are moved in the rolling mill stands with respect to their longitudinal axis. The movement rate can be fixed or time-varying. The forming/plastic deformation process takes place through the action of cylinders; based on the desired final product, the configuration of the cylinders into the rolling mill stands changes. This event is usually managed through a planned/unplanned furnace downtime [38].

#### 2.1.2. Process Automation

With regard to process automation, all reheating furnaces are equipped with automatic level 0 and level 1 managed by PLCs (Programmable Logic Controllers). In particular, level 0 includes the actuators (e.g., air/gas valves, pushers), while temperature controllers (e.g., PID-based) together with double cross-limiting strategies are located at level 1. These controllers manage the air/gas burners’ combustion reactions in the furnace zones within the different areas (see Section 1). Furthermore, at level 1, temperature controllers related to the smoke exchanger and material tracking systems are also present. Level 2 is characterized by manual, automatic, or semi-automatic systems that manage the setpoint of the level 1 controllers. Usually, level 2 systems are installed on SCADA (Supervisory Control And Data Acquisition) systems. In many steel plants, plant operators manually manage level 2 based on their experience. Level 3 is characterized by production scheduling algorithms and by systems that compute the optimal reheating profile for the billets that will enter the reheating furnace (see Section 1).

Air and fuel (e.g., natural gas) flow rates in the furnace zones are detected by flowmeters. Thermocouples measure the temperature of the furnace zones, the temperatures related to the smoke exchanger and the ambient temperature near the rolling mill. Manometers placed near the furnace inlet measure furnace and air pressures. The billets furnace’s inlet temperature is measured (usually by an optical pyrometer) only at the furnace inlet (see Figure 1). The billets furnace’s output temperature is measured (usually by optical pyrometers) after the billets furnace discharge (e.g., at the furnace outlet or in the rolling mill area, see Figure 1). Thanks to the action of the descaling devices (see Figure 1), a reliable measurement of the surface temperature of the billets can be achieved in the rolling mill area. The rolling mill’s efforts are detected through suitable measurements of the related energy absorptions and/or of the related applied forces. The rolling mill’s efforts, quantified in terms of energy absorptions and/or applied forces, strictly depend on the temperature of the billets. Suitably reheated billets allow a more convenient plastic deformation; in this way, the rolling mill’s efforts are contained. A critical aspect related to sensors in a steel reheating furnace is the lack of measurements of the temperature related to the pieces. To tackle this problem, for modelization purposes, billets temperature measurement experiments can be designed and executed using tailored test billets equipped with thermocouples, but these experiments are expensive and, furthermore, their feasibility is not always guaranteed [42,43].

#### 2.1.3. Level 2 Control and Optimization Specifications

A level 2 system designed for billets reheating furnaces must ensure correct triggering of the involved thermodynamic and physical reactions, in order to guarantee safe furnace conduction and the desired billets temperature profile. Sometimes, only a furnace discharge temperature is assigned. Safe furnace conduction means also controlling possible damages to the smoke exchanger due to too high furnace temperatures and/or gas percentages. In fact, furnace conditions may occur where dilution air is not sufficient for respecting the stringent upper constraint of the smoke exchanger temperature. The minimization of specific fuel consumption represents the crucial factor for energy efficiency achievement and improvement. In addition, there is also a need to meet stringent quality standards of the finished products and comply with stringent environmental standards (CO_2_ emissions reduction). The most challenging management requirement in a reheating furnace is the research of an optimal equilibrium between reheated billets quality and the binomial constituted by energy saving and the decreasing of the pollution’s impact, tied to specific fuel consumption minimization. In this context, energy saving can be interpreted as direct fuel saving and as indirect energy use reduction due to improvements in product quality [44,45,46].

The needed billets heating must be ensured in the furnace because the efficiency of the rolling phase is strictly related to the efficiency of the previous phase, i.e., the reheating. If the billets which enter the rolling mill stands are too cold, much higher energy consumption is required to process the billets and, furthermore, the rolling mill stands can break, leading to significant damage and maintenance costs; on the other hand, overheated billets can lead to inter-locking of the rolling mill stands and/or to excessive specific fuel consumption. The level 2 system must ensure furnace management in all process conditions: production, planned/unplanned shutdowns/downtimes, and restarts. For example, unplanned downtimes can arise due to rolling issues. The time instant of these downtimes and their duration is often unpredictable. When level 2 of the automation is represented by plant operators, due to the highly coupled multivariable, nonlinear and time-varying characteristics of the considered process, the obtained billets furnace discharge temperature is often higher than the minimum required temperature. Often, plant operators have no information about billet heating temperature profiles exhibited inside the furnace [47,48].

In order to study, design, and implement efficient level 2 systems, an accurate analysis of the considered case study is required. An example of the preliminary information required is reported in Section 2.1.4. In addition, tailored and customized structures are needed in order to select, acquire, store, analyze, and exploit data. These data structures are a key aspect in both real-time control and offline analysis (see Section 2.2.1). Furthermore, in order to compensate for the lack of information on the billet heating temperature profile exhibited inside the furnace, a virtual sensor design is another key point in the development of advanced level 2 systems for reheating furnaces (see Section 2.2.2). The process variables to be considered and the definition of control objectives are additional aspects to be addressed in a control system that is capable of handling all process conditions (see Section 2.2.3). In the present paper, the mentioned materials are combined in order to design a multi-mode MPC strategy. This strategy anticipates, when needed, some critical maneuvers (see Section 2.2.4 and Section 2.3).

#### 2.1.4. Case Study Details

In order to show an example of the performances of the proposed system, the pusher-type reheating furnace in Figure 1 and Figure 2 will be considered as a case study (see Section 3). The considered furnace is characterized by five furnace zones and six rolling mill stands. Table 1 and Table 2 report the main features of the proposed case study. The furnace production rate is typically in the range of 0–170 ton/h. In the considered furnace, before the installation of the proposed system, a yearly mean fuel consumption of about 9900 k Sm^3^ was registered. This consumption is equivalent to about 8170 toe (toe: tonne of oil equivalent).

### 2.2. Level 2 Advanced Process Control System

In this section, the proposed level 2 APC system is described, highlighting some contributions with respect to the literature. The main functional blocks of the level 2 APC system are the billets’ virtual sensor, the control mode selector, and the multi-mode MPC block.

#### 2.2.1. Data Structures

**Figure 3:**
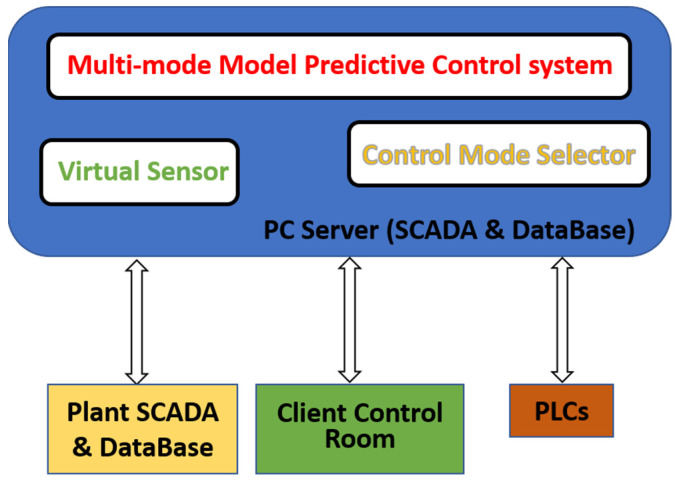
Architecture of the developed system.

In order to design a level 2 APC system for steel billets reheating furnaces, a fundamental requirement is to define efficient data structures. Selected data are acquired from the plant through PLCs. A server PC is installed on the plant and it is connected to the plant net infrastructure. The PC server hosts a SCADA system and a database. Furthermore, a client PC is installed in the control room of the reheating furnace in order to provide the selected signals information to the plant operators. A remote connection to all devices is performed in order to guarantee prompt and efficient monitoring and maintenance service. The overall APC system runs on the SCADA within the PC server [7,8]. The developed architecture is reported in Figure 3.

In the following, to describe the APC system, the pusher-type furnace described in Section 2.1 is considered. Table 3 reports an example of the information required for each billet. As can be noted from Table 3, among the billets parameters, there are geometry and thermodynamic features. Furthermore, a reheating/rolling group and a movement group are associated with each billet. Table 4 and Table 5 report an example of the parameters and the signals related to the reheating furnace and the rolling mills stands that could be used for level 2 APC purposes. SP denotes the setpoint of a control loop, while PV indicates the related process variable. The data structures can be customized based on the furnace type and the processed billets.

In Table 3, the reheating/rolling group defines the reheating profile and the rolling processing specifications for each billet. The reheating profile consists of temperature specifications (constraints and/or targets) at defined furnace/rolling mill positions and/or zone temperature settings based on the furnace conditions. The rolling processing specifications are characterized by electric current absorption constraints and/or targets. In Table 3, the movement group defines the movement parameters of the billets within the furnace and the rolling mill. Within the furnace, examples of movement parameters are the furnace movement time, the furnace exit time, the movement shift magnitude, the time to arrive at the rolling mill pyrometer from the furnace exit, and the residence time in the different furnace/rolling mill areas/zones. The movement (/furnace exit) time is defined as the time elapsed between two consecutive billet movements (/furnace exits) in the furnace. The movement shift magnitude is the length of the billet displacement related to a single furnace movement event. Within the rolling mill, movement parameters are mainly related to the speed of transport rollers and, as a consequence, to the time passed on the rolling mill stands for plastic deformation purposes.

Within the movement group settings, the movement shift magnitudes, the movement times, the furnace exit times, and other parameters (e.g., the times to arrive at the rolling mill pyrometer from the furnace exit) are updated.

#### 2.2.2. Billets’ Virtual Sensor

An online virtual sensor is designed to estimate the position, temperature, and other features of each billet, providing a billets’ map. A billet starts to be tracked when it enters the reheating furnace. Tracking stops when it leaves the rolling mill stands or if an abnormal situation occurs (e.g., inter-locking of the rolling mill stands, see Section 2.1.3). The proposed virtual sensor runs with a sampling time smaller that the sampling time of the control algorithm. Bad detection procedures (e.g., validity limits, spikes, and freezing checks) are executed on the virtual sensor-acquired inputs. Subsequently, the reheating/rolling mill parameters of each billet that is currently in the billets’ map are updated. Then, based on the updated billets’ map, the process features of the billets are updated using data from sensors (e.g., furnace zone temperatures, rolling mill ambient temperature, pyrometers measurements) and/or using suitable models (e.g., billet temperature estimation, billet rolling mill absorption estimation). Together with the billets’ map and the billet process features, the movement parameters of each group can be updated. Virtual sensor outputs to the other APC blocks are the billets’ map, the billet parameters, the movement parameters, and the temperature/absorption model’s reliability status (see the following) [12].

The online virtual sensor computes the position of each considered billet, providing an updated billets’ map. An example of a discrete-time position model that can be used for the estimation of the billet position $P_{f}$ inside the reheating furnace is the following:

(1) $$P_{f,j}\left(k+1\right)=P_{f,j}\left(k\right)+s_{f,j}\left(k\right)\;\;[m]\;P_{f,j}\left(0\right)=P_{0_{f,j}}\;\;[m]$$

where $j=1,\ldots ,m_{b}$, $m_{b}$ represents the number of billets, $P_{f,j}$ is the position of the *j*th billet, and $s_{f,j}$ is the movement shift magnitude. $P_{0_{f,j}}$ (m) is the furnace inlet position of the billet, which is computed by the virtual sensor. A model similar to that reported in Equation (1) can be applied for the billet position tracking within the rolling mill stands after the furnace discharge of the billet.

As previously described, suitable models are used to estimate and predict billet features, e.g., the temperature inside/outside the furnace and current absorption within the rolling mill stands. A discrete-time billet temperature radiation model for the estimation of the billet temperature $T_{f}$ inside the reheating furnace has been adopted [49]:

(2) $$T_{f,j}\left(k+1\right)=T_{f,j}\left(k\right)+\beta_{f,j}\left(k\right)\cdot \left(u^{4}{}_{f,j}\left(k\right)-T^{4}{}_{f,j}\left(k\right)\right)\;\;[K]\;T_{f,j}\left(0\right)=T_{0_{f,j}}\;\;[K]$$

where $k=0,\ldots ,(e_{f,j}-1)$ is the discrete time instant, $e_{f,j}$ is the furnace exit instant of the *j*th billet, $T_{f,j}$ is the mean billet temperature (K), and $u_{f,j}$ (K) is the (measured) temperature of the furnace area that hosts the considered billet. $T_{0_{f,j}}$ (K) is the mean furnace inlet temperature of the billet which is computed by the virtual sensor. $u_{f,j}$ is provided by the billets’ map. $\beta_{f,j}\left(k\right)$ coefficient is represented by:

(3) $$\beta_{f,j}\left(k\right)=\frac{T_{c}\cdot \varepsilon_{j}\left(k\right)\cdot \sigma \cdot A_{j}}{m_{j}\cdot c_{p}}\;\;[K^{-3}]$$

where $T_{c}$ (s) is the sampling time, σ (W/(m^2^·K^4^)) is the Stefan–Boltzmann constant, $A_{j}$ (m^2^) is the area of the billet exposed surface, $m_{j}$ (kg) is the mass of the *j*th billet, and $c_{p}$ (J/(kg·K)) is the specific heat of the billet. $\varepsilon_{j}\;\left(k\right)$ is the emissivity coefficient of the billet ($0\leq \varepsilon_{j}\leq 1$). Different billets can be characterized by the same emissivity coefficient (e.g., all the billets of a reheating/rolling and/or movement group, see Section 2.2.1). As it will be explained in the following, this coefficient is used to ensure robustness to uncertainties.

A discrete-time billet temperature convection model for the estimation of the billet temperature $T_{r}$ outside the reheating furnace (within the rolling mill stands) has been adopted ([49]):

(4) $$T_{r,j}\left(k+1\right)=T_{r,j}\left(k\right)+\beta_{r,j}\cdot \left(u_{r,j}\left(k\right)-T_{r,j}\left(k\right)\right)\;\;[K]\;T_{r,j}\left(0\right)=T_{f,j}\left(e_{f,j}\right)\;\;[K]$$

where $k=0,\ldots ,(e_{r,j}-1)$ is the discrete time instant, $e_{r,j}$ is the instant at which the *j*th billet leaves the considered rolling mill stands, $T_{r,j}$ is the mean billet temperature (K), and $u_{r,j}$ (K) is the (measured) temperature of the rolling mill area that hosts the considered billet. $T_{f,j}\left(e_{f,j}\right)$ is the mean furnace outlet temperature of the billet (see Equation (2)). $u_{r,j}$ is provided by the billets’ map. $\beta_{r,j}$ coefficient is represented by:

(5) $$\beta_{r,j}=\frac{T_{c}\cdot h_{j}\cdot A_{j}}{m_{j}\cdot c_{p}}\;[\;]$$

where $h_{j}$ (W/(m^2^·K)) is the convection coefficient of the billet. Different billets can be characterized by the same convection coefficient (e.g., all the billets of a reheating/rolling and/or movement group, see Section 2.2.1). The tuning of this coefficient was performed through a tailored method (not reported for brevity).

With regard to the current absorption within the rolling mill stands, a static multivariate regression model is proposed:

(6) $$a_{r,w,j}=\alpha_{0,j}+\alpha_{1,j}\cdot T_{p,j}+\alpha_{2,j}\cdot T_{0_{ff,j}}+\alpha_{3,j}\cdot r_{j}+\alpha_{4,j}\cdot T_{0_{f,j}}\;\;[A]$$

where $a_{r,w,j}$ (A) represents the mean wth current absorption of the *j*th billet ($w=1,\ldots ,n_{r})$, and $n_{r}$ is the number of rolling mill stands. $T_{p,j}$ (°C) represents a mean of the temperature values provided by the rolling mill pyrometer (see Section 2.2.1) with regard to the considered billet; this value is computed by the virtual sensor. $r_{j}$ (s) is the furnace residence time of the considered billet, i.e., the total time elapsed in the furnace; this value is computed by the virtual sensor. $T_{0_{f,j}}$ (°C) has been defined in Equation (2), while $T_{0_{ff,j}}$ (°C) is a filtered value of $T_{0_{f,j}}$. The use of the filtered input billet temperature in the regressor is motivated by the fact that the considered reheating furnace is a pusher type, where billets are attached to one another. This choice has a physical explanation: few cold billets within many hot billets benefit from the neighboring billets’ heat and, for APC purposes, can be considered as hot entering billets. Different billets can be characterized by the same $\alpha_{i,j}$ coefficients, for example, all the billets of a defined inlet temperature cluster. The tuning of the $\alpha_{i,j}$ coefficients was performed through a tailored method (not reported for brevity). The model reported in Equation (6) can be considered an innovative concept for steel industry billets reheating furnaces.

In order to ensure robustness to uncertainties, the parameter $\varepsilon_{j}\;\left(k\right)$ of Equation (3), i.e., the emissivity coefficient, can be adapted by the virtual sensor. For this purpose, a tailored optimization problem is proposed. The cost function to be minimized is:

(7) $$V_{VS}=\|T_{p}-{\hat{T}}_{p}\|{}_{Q_{p}}^{2}+\|A_{r}-{\hat{A}}_{r}\|{}_{Q_{r}}^{2}$$

subject to the following constraints:

(8) $$i.\;\;0\leq \varepsilon_{new}\leq 1\;\mathrm{ii}.\;\;lb_{d\varepsilon }\leq \varepsilon_{new}-\varepsilon_{old}\leq ub_{d\varepsilon }$$

In Equation (7), ‖·‖ indicates the Euclidean norm and $T_{p}$ $\in R^{n_{t}x1}$ contains the mean of the rolling pyrometer temperature values related to the $n_{t}$ selected billets for parameter estimation. ${\hat{T}}_{p}\in R^{n_{t}x1}$ contains the temperature estimation of each considered billet at the rolling mill pyrometer position. The temperature estimations can be expressed as a function of the $\varepsilon_{new}$ parameter, to be estimated using Equations (2)–(5). $A_{r}$ $\in R^{n_{a}x1}$ contains a set of absorption values obtained by tailored weighted means related to the mean of the rolling mill stands absorption values of the $n_{t}$ selected billets. ${\hat{A}}_{r}$ $\in R^{n_{a}x1}$ contains the absorption estimation of each of the considered $n_{a}$ weighted means. The absorption estimations can be expressed as a function of the $\varepsilon_{new}$ parameter, to be estimated using Equations (2)–(6). In Equation (7), $Q_{p}\;\in R^{n_{t}xn_{t}}$ and $Q_{r}\;\in R^{n_{a}xn_{a}}$ are suitable positive semi-definite matrixes. In Equation (8), $\varepsilon_{old}$ represents the current value of the parameter to be estimated. $\varepsilon_{new}$ is characterized by thermodynamic constraints ($0\leq \varepsilon_{new}\leq 1$) and, in addition, a lower and upper bound ($lb_{d\varepsilon }$, $ub_{d\varepsilon }$) were introduced in order to limit the innovation on the estimated parameter. The cost function reported in Equation (7) takes into account both rolling mill pyrometer feedback and rolling mill stands’ absorption feedback. The computed $\varepsilon_{new}$ coefficient updates the $\varepsilon_{j}\;\left(k\right)$ coefficient of the billets of the considered group. This coefficient is used in the billets’ temperature computation (see Equations (2) and (3)).

The optimization problem for the emissivity coefficient adaptation, represented by Equations (7) and (8), can be solved using different techniques. For example, for scalar parameter estimation, MATLAB fminbnd function can be used, based on golden section search and parabolic interpolation [50].

The parameters adaptation method can be suitably customized for each type of billet model and for each type of temperature/absorption/force sensor. Furthermore, if a case study requires differentiation between the head and the tail (see Section 2.1.1) billet model, suitable processing of rolling pyrometer measurements is needed. The developed parameters adaptation algorithm runs in parallel with respect to the other procedures of the virtual sensor. To the best of the authors’ knowledge, the proposed optimization problem is innovative in the field of level 2 APC systems for steel industry billets reheating furnaces.

In order to evaluate the efficiency of the used temperature/absorptions models, the virtual sensor uses customized thresholds which are used for validation when updated measurements are available, before the execution of the described adaptation procedure. In this way, temperature/absorption reliability status is computed based on the feedback information. Furthermore, the reliability of the temperature models is also checked through thresholds on the difference between the billet temperature estimations with and without parameter adaptation. In addition, the reliability of the absorption models requires the reliability of the temperature models (as a consequence of Equation (6)).

#### 2.2.3. APC Variables and Objectives

In order to design the level 2 APC system, a basic structure related to Manipulated Variables (MVs), measured Disturbance Variables (DVs), and CVs is reported.

Usually, the MVs are the temperature setpoints related to the level 1 controllers (see Section 2.1.2). MVs are usually characterized by hard constraints on the magnitude and on the allowed variation in the defined sampling time [51,52]. Furthermore, as will be described in Section 2.3, a target can be imposed. The main DVs are represented by the billets’ furnace inlet temperature and by the movement parameters, i.e., furnace movement time, furnace exit time, movement shift magnitude, and the time to arrive at the rolling mill pyrometer from the furnace exit (see Section 2.2.4).

Table 6 reports the main CVs and the related priorities (a lower number corresponds to a higher priority). $T_{f,j,P_{f,j,i}}$ represents the temperature in the furnace position $P_{f,j,i}$ ($i=1,\ldots ,m_{t,f,j}$) of the *j*th billet that has to be controlled. $m_{t,f,j}$ represents the total number of furnace positions subjected to temperature control specifications on *j*th billet. $A_{r,j,c}$ ($c=1,\ldots ,m_{a,r,j}$) represents weighted means of absorptions variables related to the *j*th billet to be controlled. $m_{a,r,j}$ represents the number of rolling mill absorption weighted means subjected to control specifications on *j*th billet. These means are named rolling mill loads. $\Delta T_{f,j,P_{f,j,d}}$ represents the temperature difference between the tail and the head of the *j*th billet that has to be controlled in the furnace position $P_{f,j,d}$ ($d=1,\ldots ,m_{\Delta t,f,j}$). $m_{\Delta t,f,j}$ represents the number of furnace positions subjected to temperature difference control specifications on *j*th billet. The control of $\Delta T_{f,j,P_{f,j,d}}$ can be performed in two different ways: through a tailored billet temperature difference model or through different management of MVs that separately act on the head and of the tail of the billets (e.g., equalization described in Section 2.1.1). The just described CV types can be characterized by targets and/or *soft* constraints [51,52]. Other main CVs are represented by the smoke exchanger temperature (see Section 2.1.3) that is upper constrained and by additional variables, e.g., gas valves. These additional variables, through their upper and/or lower constraints, can support the level 2 controller, in particular, furnace conditions. For example, in a furnace condition characterized by a billet charge with different inlet temperatures, some zones can be subjected to excessive gas valves opening, which may reveal not optimal furnace conduction.

The reported APC variables structure can be customized based on the single case study, for example introducing additional DVs, e.g., the furnace/air pressure. Furthermore, MVs can also be represented by gas/air flow rates and stoichiometric ratios setpoints: in these cases, some CVs need to be added, for example, the zone temperatures process variables [12,47,48,53].

The proposed choice of CVs (see Table 6) and the double option on billet head–tail temperature difference are innovative in the field of level 2 APC systems.

In many plants, $T_{f,j,P_{f,j,i}}$ targets and/or constraints are not known beforehand by engineers/operators (e.g., quality department). Furthermore, the application of level 3 approaches is not always feasible. Often. billet temperature measurement experiments are not feasible, so a reliable specifications definition can be performed only in the position of the available sensors, e.g., the rolling mill pyrometer. In order to achieve the specifications at the furnace discharge position, Equations (4) and (5) can be solved backwards. The just proposed specifications computation procedure is innovative in steel industry billets reheating furnaces.

#### 2.2.4. Furnace Condition Detection and Multi-Mode MPC Definition

As described in Section 2.1.1, different furnace conditions can occur: production, shutdowns, downtimes, and restarts. Using the acquired and computed data of the virtual sensor, and also exploiting the information provided by operators, a control mode selector is proposed which identifies the current and future furnace conditions. The selector is characterized by a sampling time equal to the MPC sampling time (see the following). The high variability of the process considered, together with the need for a system capable of handling all process conditions 24/7, motivated the effort to study, design, and implement a control mode selector.

The operators can provide different information to the control mode selector:

- communication of a condition change, e.g., the instantaneous start of an unplanned downtime/shutdown;
- communication of an expected condition change, e.g., declaring the last billet on the billets’ map to be exited from the furnace before a planned downtime/shutdown or the remaining time before a planned downtime. Furthermore, the production restart timestamp can be communicated by plant operators.

The furnace condition can significantly influence the control mode based on the specific case study. For example, if the developed billet temperature and/or absorption model (see Section 2.1.2) is not reliable in conditions that are different from the production one, the considered model cannot be used. In addition, in order to choose online the best control mode in each process condition, an additional check (preview status) is performed to evaluate if predictions on furnace operability can be reliable: in some cases, high variability of process variables prevents the full MPC paradigm to be applied.

The estimated movement parameters are updated using the virtual sensor outputs and operators’ information (e.g., the remaining time before a planned downtime). The proposed approach can also be extended if the information on the billets that will be charged in the furnace is available.

Depending on the detected current furnace condition (production, planned/unplanned shutdowns/downtimes, and restarts), the reliability status computed by the virtual sensor, and the preview status, the best control mode is chosen online by the selector. Suitable hysteresis logics were introduced in order to avoid chattering within the control mode selection. Using this approach, a multi-mode MPC approach is designed (see formulation in Section 2.3): Table 7 shows the control modes’ activation matrix, while Table 8 reports the CVs involved in each control mode. Note that, thanks to the estimated movement parameters, the level 2 control specifications in selected plant positions (associated to $T_{f,j,P_{f,j,i}}$ and $\Delta T_{f,j,P_{f,j,d}}$ CVs, see Table 6) are rearranged as control specifications on the prediction horizon.

As can be observed in Table 7 and Table 8, the control mode 3 includes a major number of CVs; in particular, weighted means of absorptions variables related to the *j*th billet to be controlled ($A_{r,j,c}$ in Table 8) are present only in this control mode. The control mode 2 does not include the $A_{r,j,c}$ CVs, but, with respect to control mode 1, it takes into account billets temperature CVs ($T_{f,j,P_{f,j,i}}$ in Table 8). Other CVs of Table 8, e.g., the smoke exchanger temperature, are included in the formulation of all control modes. In addition, control modes 2–3 require a reliable preview status (see Table 7).

An MPC horizons adaptation methodology is used (see Section 2.3) [53]. In particular, thanks to the estimated movement parameters, a prediction horizon for each billet to be considered is computed. The ideal prediction horizon of the *j*th billet represents its remaining furnace residence time, but, due to the previously described process features, only an estimation of this residence time can be achieved at the kth instant, i.e., ${\hat{H}}_{p,j}(k|k)$. The MPC prediction horizon $H_{p}\left(k\right)$ is the maximum among ${\hat{H}}_{p,j}(k|k)$.

The proposed multi-mode MPC approach can be used for different furnace types (e.g., walking beam and pusher type) regardless of the adopted billet models. Furthermore, it manages all process conditions and it can be suitably customized based on the defined control specifications. To the best of the authors’ knowledge, the proposed control mode selector and the multi-mode MPC approach are innovative in the field of level 2 APC systems for steel industry billets reheating furnaces.

### 2.3. Multi-Mode MPC Formulation

The present section details the three control modes that are proposed (see Section 2.2.4), highlighting some contributions with respect to the literature.

#### 2.3.1. General Formulation and Control Modes Characterization

A general level 2 MPC problem is proposed which is suitably customized for each control mode. The cost function to be minimized is:

(9) $$\begin{array}{cc} V_{MPC}\left(k\right) & =\overset{H_{p}\left(k\right)-1}{\underset{i=0}{\sum }}{\left\|\hat{u}\left(k+i|k\right)-u_{t}\left(k+i|k\right)\right\|}_{S\left(i\right)}^{2}\\ & +\overset{H_{p}\left(k\right)}{\underset{i=1}{\sum }}{\left\|\hat{y}\left(k+i|k\right)-y_{t}\left(k+i|k\right)\right\|}_{Q\left(i\right)}^{2}+\overset{H_{u}\left(k\right)}{\underset{i=1}{\sum }}{\left\|\Delta \hat{u}\left(k+M_{i}\left(k\right)|k\right)\right\|}_{ℛ\left(i\right)}^{2}\\ & +\overset{m_{b}}{\underset{j=1}{\sum }}\overset{{\hat{H}}_{p,j}\left(k|k\right)}{\underset{i=1}{\sum }}q_{T,j,i}\cdot {\left({\hat{T}}_{f,j}\left(k+i|k\right)-T_{T,f,j}\left(k+i|k\right)\right)}^{2}\\ & +\overset{m_{b}}{\underset{j=1}{\sum }}\overset{{\hat{H}}_{p,j}\left(k|k\right)}{\underset{i=1}{\sum }}q_{\Delta T,j,i}\cdot {\left({\hat{\Delta T}}_{f,j}\left(k+i|k\right)-\Delta T_{T,f,j}\left(k+i|k\right)\right)}^{2}\\ & +\overset{m_{b}}{\underset{j=1}{\sum }}\overset{m_{a,r,j}}{\underset{i=1}{\sum }}q_{A,j,i}\cdot {\left({\hat{A}}_{r,j,i}-A_{t,r,j,i}\right)}^{2}+{\left\|\varepsilon_{y}\left(k\right)\right\|}_{\rho_{\varepsilon_{y}}}^{2}\\ & +{\left\|\varepsilon_{T}\left(k\right)\right\|}_{\rho_{\varepsilon_{T}}}^{2}+{\left\|\varepsilon_{\Delta T}\left(k\right)\right\|}_{\rho_{\varepsilon_{\Delta T}}}^{2}+{\left\|\varepsilon_{A}\left(k\right)\right\|}_{\rho_{\varepsilon_{A}}}^{2} \end{array}$$

subject to the following constraints:

(10) $$i.\;lb_{du}\leq \Delta \hat{u}(k+M_{i}\left(k\right)|k)\leq ub_{du},i=1,\;\ldots ,\;H_{u}\left(k\right)\;\mathrm{ii}.\;lb_{u}\left(i\right)\leq \hat{u}(k+M_{i}\left(k\right)|k)\leq ub_{u}\left(i\right),i=1,\;\ldots ,\;H_{u}\left(k\right)\;\mathrm{iii}.\;lb_{y}\left(i\right)-\gamma_{lby}\left(i\right)\cdot \varepsilon_{y}\left(k\right)\leq \hat{y}\left(k+i|k\right)\leq ub_{y}\left(i\right)+\gamma_{uby}\left(i\right)\cdot \varepsilon_{y}\left(k\right),\;i=1,\ldots ,\;H_{p}\left(k\right)\;\mathrm{iv}.\;lb_{T}\left(i\right)-\gamma_{lbT}\left(i\right)\cdot \varepsilon_{T}\left(k\right)\leq {\hat{T}}_{f,j}\left(k+i|k\right)\leq ub_{T}\left(i\right)+\gamma_{ubT}\left(i\right)\cdot \varepsilon_{T}\left(k\right),\;j=1,\ldots ,\;m_{b}\;\;\;\;\;i=1,\ldots ,\;{\hat{H}}_{p,j}(k|k)\;v.\;lb_{\Delta T}\left(i\right)-\gamma_{lb\Delta T}\left(i\right)\cdot \varepsilon_{\Delta T}\left(k\right)\leq {\hat{\Delta T}}_{f,j}\left(k+i|k\right)\leq ub_{\Delta T}\left(i\right)+\gamma_{ub\Delta T}\left(i\right)\cdot \varepsilon_{\Delta T}\left(k\right),\;j=1,\ldots ,\;m_{b}\;\;\;\;\;i=1,\ldots ,\;{\hat{H}}_{p,j}(k|k)\;\mathrm{vi}.\;lb_{A}\left(i\right)-\gamma_{lbA}\left(i\right)\cdot \varepsilon_{A}\left(k\right)\leq {\hat{A}}_{r,j,i}\leq ub_{A}\left(i\right)+\gamma_{ubA}\left(i\right)\cdot \varepsilon_{A}\left(k\right),\;j=1,\ldots ,\;m_{b}\;\;\;\;\;i=1,\ldots ,\;m_{a,r,j}\;\mathrm{vii}.\;\varepsilon_{y}\left(k\right)\geq 0;\varepsilon_{T}\left(k\right)\geq 0;\varepsilon_{\Delta T}\left(k\right)\geq 0;\varepsilon_{A}\left(k\right)\geq 0$$

In Equation (9), the Euclidean norm is used. The cost function reported in Equation (9) and the *hard/soft* constraints reported in Equation (10) represent an MPC formulation with tracking, move suppression, and slack variables terms [54,55]. In Equations (9) and (10), $u\in R^{l_{u}x1}$ represent the MVs vector, while $y\in R^{m_{y}x1}$ represents the CVs vector, which refers to the last two groups of CVs of Table 6. Furthermore, the slack variable vectors $\varepsilon_{y}\left(k\right)$, $\varepsilon_{T}\left(k\right)$, $\varepsilon_{\Delta T}\left(k\right)$ and $\varepsilon_{A}\left(k\right)$ can be characterized by different elements, based on the case study. MV tracking terms ($u_{t}\left(k+i|k\right)$ in Equation (9)) can be used for MVs minimization. These terms can be provided by another optimizer (see, e.g., [47,48]) or by a tailored computation algorithm.

Smoke exchanger temperature and gas valves CVs (see Table 6) can be characterized by First-Order Plus Deadtime (FOPDT) linear models [56]. For these variables, model mismatch compensation can be obtained through a deadbeat Kalman filter [57,58]. Zone temperature PVs can also be used as intermediate variables between MVs and the billet temperature model [48,53].

In Equations (9) and (10), $H_{u}\left(k\right)$ is the control horizon, while the $M_{i}\left(k\right)$ terms represent the input move blocking instants [59]. In order to obtain tractable optimization problems, the control horizon $H_{u}\left(k\right)$ is not set equal to the prediction horizon $H_{p}\left(k\right)$, but the following formula is used:

(11) $$H_{u}\left(k\right)=ceil\left(\frac{H_{p}\left(k\right)}{r_{H_{p}-H_{u}}}\right)$$

where $r_{H_{p}-H_{u}}$ represents the desired ratio between $H_{p}\left(k\right)$ and $H_{u}\left(k\right)$. Accordingly, an online adaptation law is defined also for $M_{i}\left(k\right)$ ($i=1,\;\ldots ,\;H_{u}\left(k\right)$) instants, based on the adapted values of $H_{p}\left(k\right)$ and $H_{u}\left(k\right)$. The authors proposed the following online adaptation law (see [53]):

(12) $$M_{1}=0\;M_{2}=1\;\vdots \;M_{\mu }=\mu -1\;M_{\mu +1}=\mu +\alpha \;M_{\mu +2}=\mu +\alpha +\beta \;M_{\mu +3}=\mu +\alpha +2\cdot \beta \;\vdots \;M_{H_{u}}=\left(\mu +\alpha \right)+\left(H_{u}-\left(\mu +\alpha \right)\right)\cdot \beta$$

where:

(13) $$\mu \leq H_{u};\;\;\;\;\;\;\;\;\;\;\;\;\;\;\alpha \in \mathbb{N};\;\beta =fix\left(\frac{\left(H_{p}-\omega \right)-\left(\mu +\alpha \right)}{\left(H_{u}-\mu \right)}\right);\;\omega \in \mathbb{N}$$

In Equation (12), fix(x) rounds x to the nearest integer less than or equal to x. The adaptation law reported in Equations (12) and (13) defines the $M_{i}\left(k\right)$ prediction instants related to the MVs future moves in order to ensure several MVs control moves in the initial prediction instants and to ensure a sufficient control horizon [53].

Based on computed $M_{i}\left(k\right)$ instants (see Equations (12) and (13)), on the computed control horizon (see Equation (11)) and on the computed prediction horizon (see Section 2.2.4), the MVs predictions of Equations (9) and (10) are parametrized as follows [53,54]:

(14) $$\hat{u}\left(k+M_{1}\left(k\right)|k\right)=\ldots =\hat{u}\left(k+M_{2}\left(k\right)-1|k\right)=u\left(k-1\right)+\Delta \hat{u}(k+M_{1}\left(k\right)|k)\;\hat{u}\left(k+M_{2}\left(k\right)|k\right)=\ldots =\hat{u}\left(k+M_{3}\left(k\right)-1|k\right)=u(k+M_{1}\left(k\right)|k)+\Delta \hat{u}(k+M_{2}\left(k\right)|k)\;\vdots \;\hat{u}\left(k+M_{H_{u}}\left(k\right)|k\right)=\ldots =\hat{u}\left(k+H_{p}\left(k\right)-1|k\right)=\hat{u}(k+M_{H_{u}-1}\left(k\right)|k)+\Delta \hat{u}(k+M_{H_{u}}\left(k\right)|k)$$

The formulation reported in Equations (9) and (10) is nonlinear if the model reported in Equation (2) is used. A Quadratic Programming (QP) problem is obtained in Equations (9) and (10) through the following linearization of Equation (2):

(15) $${\hat{T}}_{f,j}\left(k+i|k\right)={\hat{T}}_{f,j}\left(k+i-1|k\right)+{\hat{\beta }}_{f,j}\left(k|k\right)\cdot \left({\hat{u}}^{2}{}_{f,j,free}\left(k+i-1|k\right)+{\hat{T}}^{2}{}_{f,j,free}\left(k+i-1|k\right)\right)\cdot \;\left({\hat{u}}_{f,j,free}\left(k+i-1|k\right)+{\hat{T}}_{f,j,free}\left(k+i-1|k\right)\right)\cdot ({\hat{u}}_{f,j}\left(k+i-1|k\right)-\;{\hat{T}}_{f,j}\left(k+i-1|k\right))\;\;[K]\;{\hat{T}}_{f,j}\left(k|k\right)={\hat{T}}_{k_{f,j}}\;[K]\;\;\;\;\;\;\;\;\;\;\;\;\;\;\;j=1,\ldots ,\;m_{b}\;\;\;\;\;i=1,\ldots ,\;{\hat{H}}_{p,j}(k|k)$$

where ${\hat{T}}_{k_{f,j}}$ represents the temperature estimation at the current instant k and the subscript *free* represents the *free response* at the current instant k. The *free response* is defined as the prediction considering the previous input values u(k−1) as input, thus assuming $\Delta \hat{u}\left(k+M_{i}\left(k\right)|k\right)=0$ [54]. The model reported in Equation (15) is a Linear Parameter Varying (LPV) model [60]. The obtained QP problem based on Equations (1)–(15) can be solved using different techniques: for example, MATLAB quadprog solver can be used [50].

To the best of the authors’ knowledge, the proposed linearization approach and the LPV formulation are innovative in the field of level 2 APC systems for steel industry billets reheating furnaces.

Based on the selected control mode (see Section 2.2.4), the CVs reported in Table 8 are included in the MPC formulation for each control mode. With regard to control mode 1, as can be noted in Table 8, CVs related to billet temperature and absorption are not considered. In order to control the furnace, a tailored policy for MVs targets ($u_{t}\left(k+i|k\right)$) definition in Equation (9) is proposed (not reported for brevity). As described in Section 2.2.1, reheating/rolling group could include zone temperature settings based on the furnace condition. In this way, temperature setpoint minimization is performed while avoiding overheating or underheating the billets within each furnace area.

## 3. Results and Discussion

In the present section, an example of the performance of the proposed APC system is shown. The reheating furnace described in Section 2.1.4 is considered as case study for field results description. Modelling and control results associated with the level 2 process control will be shown, together with some results on the energy efficiency improvement.

### 3.1. Level 2 Modelization Results

**Figure 4:**
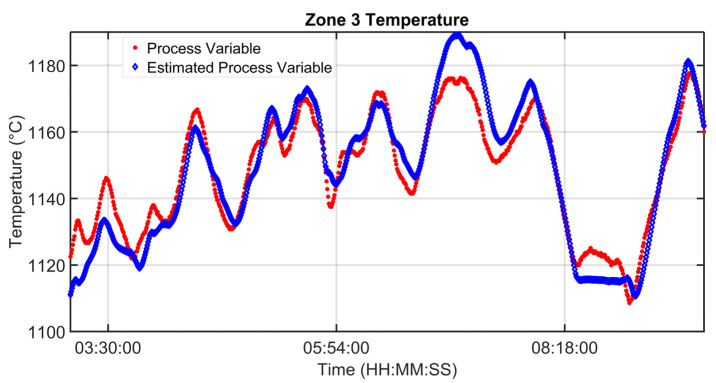
Modelization results: zone 3 temperature PV model.

**Figure 5:**
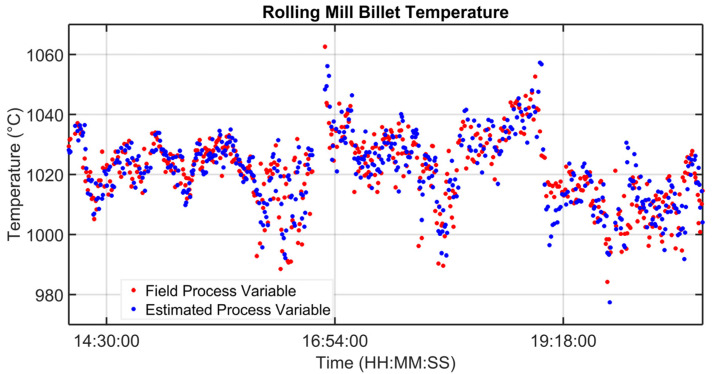
Modelization results: billets temperature model.

**Figure 6:**
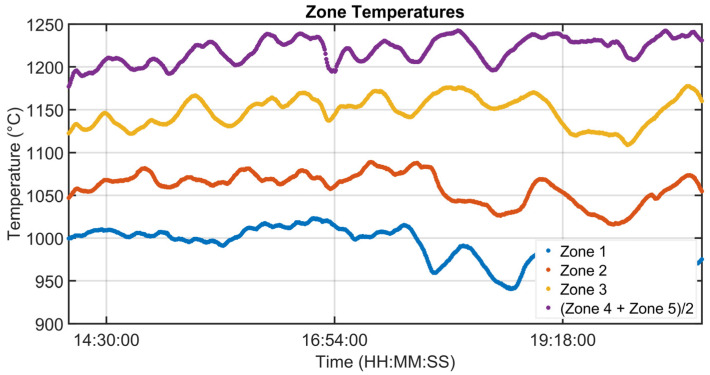
Modelization results: zone temperatures associated with the billets temperature model.

**Figure 7:**
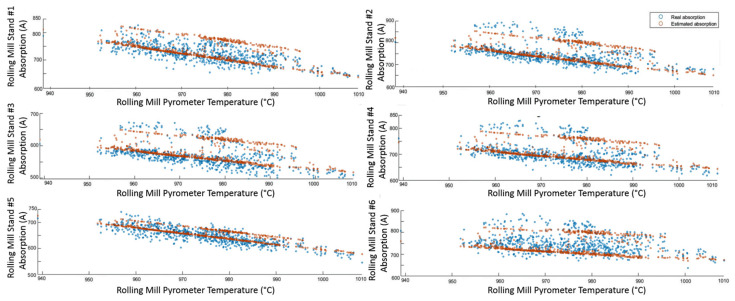
Modelization results: billets rolling mill stand absorption model.

Since the proposed APC system is based on a model-based control strategy, the modelling phase plays a fundamental role. Given the remarkable modelling results achieved, the adoption of an MPC strategy was justified. Figure 4, Figure 5, Figure 6 and Figure 7 report some modelling results. Figure 4 reports the performance of the zone 3 temperature PV model over a period of about six hours. This model allows us to use zone 3 temperature’s PV as an intermediate variable between the MVs and billets temperature model (see Section 2.3.1). Figure 5 depicts the billets’ temperature at a rolling mill pyrometer (red dots) and its estimation (blue dots) during a period of about six hours. In Figure 5, each dot is associated with a billet. Inputs related to this model are reported in Figure 6. Analyzing different years’ process data, a Root Mean Square Error of Prediction (RMSEP) less than 10 °C has been obtained (about 1% of the optical pyrometers measurement range). Figure 7 reports current absorption model results. Absorption predictions (orange) computed by the multivariate regression model (see Equation (6)) are compared with real data (blue). The rolling mill load value used for control purposes (see Section 2.2.3) is computed as a weighted mean of the current absorptions. Observing the reported results, it can be noticed that the obtained models guarantee an acceptable approximation of the behavior of the considered variables.

### 3.2. Level 2 Control Results

**Figure 8:**
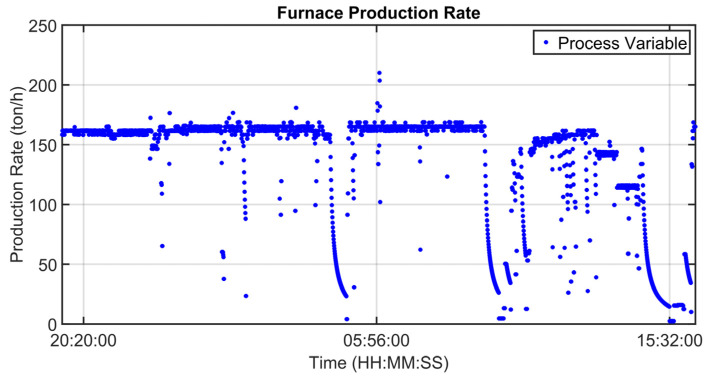
Control results: furnace production rate.

**Figure 9:**
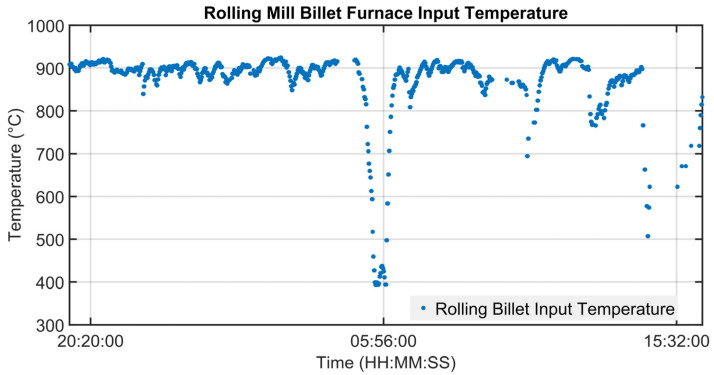
Control results: billets temperature at furnace inlet pyrometer.

**Figure 10:**
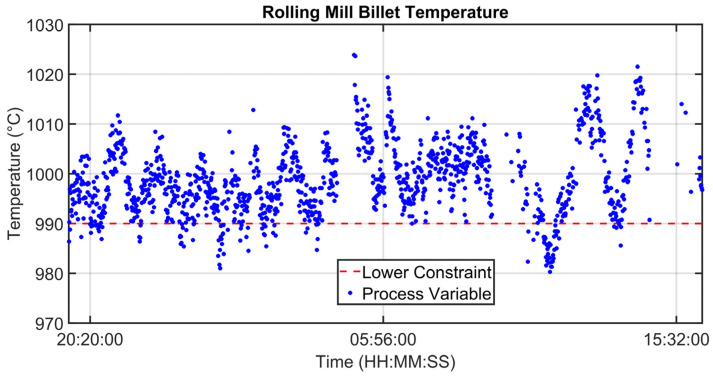
Control results: billets temperature at rolling mill pyrometer.

**Figure 11:**
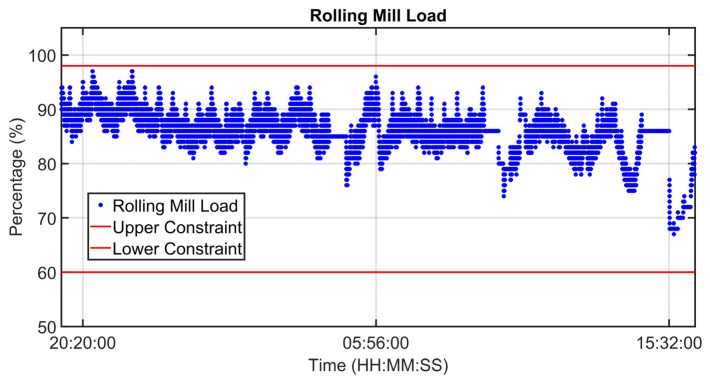
Control results: billets rolling mill load.

**Figure 12:**
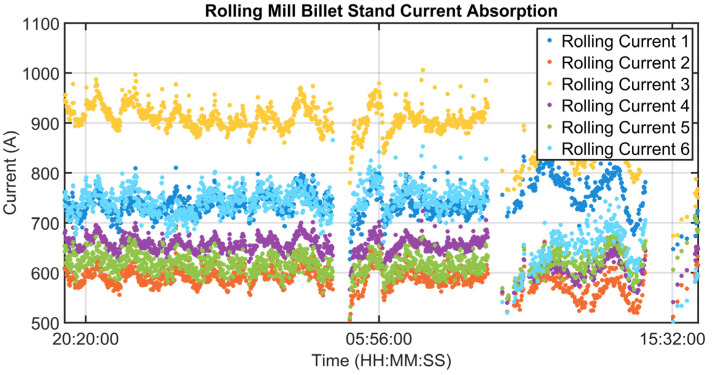
Control results: rolling mill stand absorptions.

**Figure 13:**
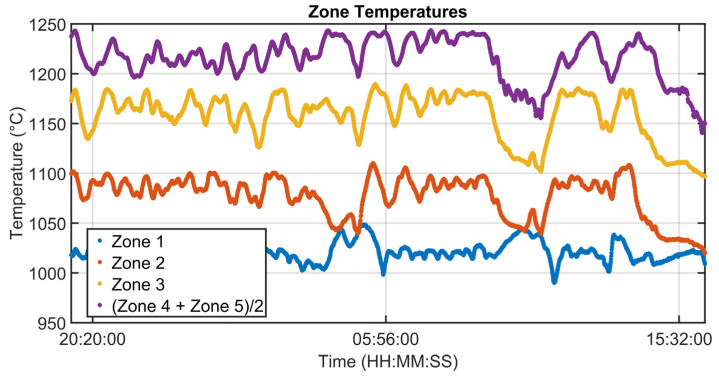
Control results: zone temperatures.

Figure 8, Figure 9, Figure 10, Figure 11, Figure 12 and Figure 13 show an example of level 2 control performances during a period of about twenty hours. In the proposed period, a not constant furnace production rate is registered (see Figure 8) together with a varying billets furnace inlet temperature (see Figure 9). The furnace production rate is computed through the measured movement parameters, while the furnace inlet temperature of each billet is computed through a tailored procedure which uses optical pyrometer data. The furnace production rate and the billets furnace inlet temperature represent two significant DVs for the APC system. With regard to the furnace production rate, production and downtimes operating conditions can be observed in Figure 8. In the production operating condition, the furnace production rate reaches about 160 ton/h. With regard to the furnace inlet temperature, this process variable varies in the range of 400–900 °C (see Figure 9). 

Although the furnace production rate and inlet temperature are not constant, the APC system guarantees that constraints on rolling mill temperature and rolling mill load are met (see Figure 10 and Figure 11). In Figure 10 and Figure 11, constraints are depicted through red lines, while process variables are represented through blue lines. The rolling mill stands absorptions used for the computation of the rolling mill load are reported in Figure 12. A different color is associated with the absorption of each rolling mill stand. The zone temperatures PVs are reported in Figure 13: they represent the PVs associated with the SPs manipulated by the APC system. The APC system, thanks to the designed multi-mode MPC strategy, smartly manipulates the zone temperature setpoints in order to respect the level 2 control specifications and to save energy as much as possible. Observing Figure 10, it can be noticed that the temperature lower constraint is targeted by the APC system. The reported violations of the lower constraint are negligible. In this way, energy efficiency maximization is achieved while ensuring the required level 2 control specifications. The designed APC system is able to compensate for the not constant DVs (see Figure 8 and Figure 9) thanks to the developed multi-mode MPC strategy. This strategy allows us to handle and optimize all process conditions.

**Figure 14:**
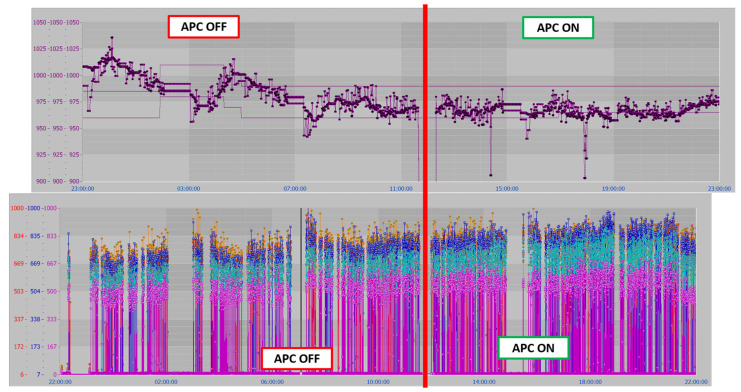
Control results: pre/post-APC system comparison (billets temperature at the rolling mill and rolling mill stands current absorptions).

Figure 14 reports a pre-post APC comparison. The first plot (on the top of the figure) reports the temperature of the billet measured by the rolling mill pyrometer and the associated constraints. The second plot (on the bottom of the figure) shows the rolling mill stands absorptions measured in the considered period; here, different colors refer to different stands. In the left part of the reported plots, the APC system is not active: as can be observed in the plot on top of Figure 14, the constraints are not always met and, in some cases, overheating occurs. As a consequence, the rolling mill stands absorptions are very low (see bottom of Figure 14). As can be noted, the APC system improves the billet’s temperature control performances and optimizes the process through temperature minimization (see right side on the top of Figure 14). A consequent current absorptions increase is observed which is acceptable (see right side at the bottom of Figure 14).

### 3.3. Level 2 Energy Efficiency Results

Different installations on European real plants confirmed the validity and flexibility of the proposed level 2 control approach. Energy efficiency and process control performances were significantly improved with respect to operators’ manual conduction and/or previous level 2 control systems. Year-specific fuel consumption reduction in the range of 2–6% was obtained, together with a service factor greater than 90% in all applications. The APC system received an energy efficiency award and the Industry 4.0 certification.

**Figure 15:**
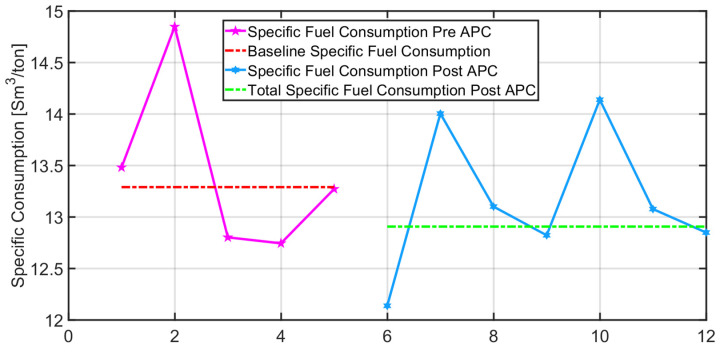
Control results: pre/post-APC system comparison on specific fuel consumption considering different production periods.

In order to certify the energy efficiency benefits provided by the APC system, specific fuel consumption was considered as a Key Performance Indicator (KPI). Taking into account the pusher-type reheating furnace described in Section 2.1.4, the specific fuel consumption related to selected production periods has been evaluated. A baseline has been computed, considering the production periods before the installation of the APC system. For the baseline computation, the most significant data that have been considered are: the amount of steel processed in the furnace, the amount of final product, billets furnace inlet temperature, hot charge percentage, and current absorption of the rolling mill stands. In this way, the not significant production periods could be discarded (“outlier”) from the KPI computation. For example, considering a selected final product, five production periods before the APC system installation (Figure 15, magenta) and seven production periods after the APC system installation (Figure 15, light blue) have been selected. Based on the selected periods before APC system installation, the baseline has been computed (Figure 15, red). The cumulative specific fuel consumption after the APC system installation (Figure 15, green) has been decreased by 2.88% with respect to the computed baseline. This approach for energy efficiency assessment, based on a baseline computation, has been used for all production periods related to the most significant final products. In this way, a specific fuel consumption reduction of around 2% has been certified for the considered furnace. Considering a yearly mean fuel consumption of about 9900 k Sm^3,^ which is equivalent to about 8170 toe (tons of oil equivalent), the obtained energy saving is highly evaluable.

## 4. Conclusions

In this paper, a unified level 2 APC framework for steel billets reheating furnaces is proposed. The topic is of great interest in the field, as proven by the extensive literature available in recent years. Compared with the known state of the art, the control system proposed in the present work is primarily characterized by its capability to efficiently handle all operating conditions, such as furnace downtimes and highly varying scenarios. In this way, optimized management is always ensured. The proposed control framework is equipped with customization procedures for each different furnace plant, e.g., walking beam and pusher type. The APC system is characterized by a billets’ virtual sensor, a control mode selector, and a multi-mode MPC strategy. Different state-of-art innovative features were added. The provided novelties refer to modelling methods, control matrix definition, and the control strategy for level 2 APC systems on steel billets reheating furnaces. The research work achieved an energy efficiency award together with Industry 4.0 certification.

Different installations on European real plants confirmed the validity and flexibility of the proposed level 2 control approach. Significant improvements with respect to operators’ manual conduction and/or previous level 2 control systems were achieved in terms of energy efficiency and process control performances. The features of the proposed unified level 2 APC framework allowed us to manage all process conditions in an optimized manner, providing remarkable control results while, at the same time, targeting significant energy efficiency results. Year-specific fuel consumption reduction in the range of 2–6% was obtained, together with a service factor greater than 90% in all applications.

Future work will be focused on research on further methods for level 2 control systems to be applied to steel industry reheating furnaces. In addition, controllers at other automation levels will be investigated. Finally, some studies about other subprocesses of the steel industry will be conducted in order to extend the application areas of the designed control systems.

## Figures and Tables

**Table 1:** Billet features.

Feature	Value
Height	0.14–0.15 [m]
Width	0.14–0.15 [m]
Length	12.2 [m]
Mass	1.87–2.155 [ton]
Inlet Temperature	30–910 [°C]
Desired Rolling Mill Temperature	950–1070 [°C]
Desired Rolling Mill Load	60–98 [%]

**Table 2:** Furnace zones features.

Furnace Zone	Billets Number	Temperature Setpoint Range
Zone 1	35–38	825–1020 [°C]
Zone 2	37–40	925–1090 [°C]
Zone 3	31–33	1025–1180 [°C]
Zone 4–5	25–27	1115–1240 [°C]

**Table 3:** Example of billets information.

Parameter Type	Parameter	Measurement Unit
Geometry	Length	[m]
	Height	[m]
	Width	[m]
	Mass per unit length	[kg/m]
Thermodynamic	Specific heat	[J/(kg∙K)]
	Thermal conductivity	[W/(m∙K)]
	Convection coefficient	[W/(m^2^∙K)]
	Emissivity coefficient	[ ]
	Furnace inlet temperature	[K]
	Furnace inlet temperature filtered	[K]
Reheating/Rolling	Reheating/Rolling group	[ ]
Movement	Movement group	[ ]

**Table 4:** Furnace parameters.

Parameter Group	Parameter	Measurement Unit
Furnace	Length of each zone	[m]
	Position of the furnace zones thermocouples	[m]
	Start position of each zone in the furnace	[m]
	Minimum position for billet discharge	[m]

**Table 5:** Furnace/rolling mill signals.

Parameter Group	Parameter	Signal Type/Measurement Unit
Furnace	Inlet photocell	Boolean/[ ]
	Billet charged trigger	Boolean/[ ]
	Outlet photocell	Boolean/[ ]
	Inlet pyrometers	Real/[°C]
	Zone temperatures (SP, PV)	Real/[°C]
	Smoke exchanger temperature	Real/[°C]
	Zones gas/air flow rate (SP, PV)	Real/[Nm^3^/h]
	Outlet pyrometer	Real/[°C]
Rolling Mill	Inlet photocell	Boolean/[ ]
	Stands Absorption	Real/[A]
	Ambient temperature	Real/[°C]
	Rolling mill pyrometer	Real/[°C]

**Table 6:** APC main CVs.

Variable Name	Measurement Unit	Priority
$T_{f,j,P_{f,j,i}}$	[°C]	2
$A_{r,j,c}$	[A] (or [%])	2
$\Delta T_{f,j,P_{f,j,d}}$	[°C]	2
Smoke exchanger temperature	[°C]	1
Gas valves	[%]	TUNABLE

**Table 7:** Control modes activation matrix.

Status	Control Mode 1	Control Mode 2	Control Mode 3
Furnace conditions status	Variable	Variable	Variable
Absorption model reliability status	0	0	1
Temperature model reliability status	0	1	1
Preview status	0	1	1

**Table 8:** Control modes characterization (CVs).

Variable Name	Control Mode 1	Control Mode 2	Control Mode 3
$T_{f,j,P_{f,j,i}}$		X	X
$A_{r,j,c}$			X
$\Delta T_{f,j,P_{f,j,d}}$	X	X	X
Smoke exchanger temperature	X	X	X
Gas valves	X	X	X

## Data Availability

Not applicable.
